# Cytotoxicity and Mineralization Activity of Calcium Silicate-Based Root Canal Sealers Compared to Conventional Resin-Based Sealer in Human Gingival Fibroblast Cells

**DOI:** 10.1155/2023/4376579

**Published:** 2023-05-31

**Authors:** Mohammad Shokrzadeh, Farzaneh Sadat Motafeghi, Anahita Lotfizadeh, Mohammad Ghorbani, Azam Haddadi Kohsar

**Affiliations:** ^1^Department of Toxicology-Pharmacology, Faculty of Pharmacy, Mazandaran University of Medical Sciences, Sari, Iran; ^2^Student Research Committee, Faculty of Dentistry, Mazandaran University of Medical Sciences, Sari, Iran; ^3^Dentist, Sari, Mazandaran, Iran; ^4^Department of Endodontics, Dental Research Center, Faculty of Dentistry, Mazandaran University of Medical Sciences, Sari, Iran

## Abstract

**Background:**

Root canal obturation is performed by gutta-percha cones and sealer. Therefore, these materials, specially sealers, must be biocompatible. This study investigated the cytotoxicity and mineralization activity of two calcium silicate-based sealers (Endoseal MTA and Ceraseal) and an epoxy resin-based sealer (AH26).

**Materials and Methods:**

In this experiment, the cytotoxicity of Endoseal MTA, Ceraseal, and AH26 on human gingival fibroblast cells was examined using Methyl-Thiazol-Tetrazolium assay at time intervals of 24, 48, 72, and 120 hr. The mineralization activity of sealers was evaluated by Alizarin red staining assay. Prism, ver.3, software was used to perform statistical tests. One-way analysis of variance analysis, followed by Tukey's test, was used to determine the group differences. *P*-values < 0.05 were considered statistically significant.

**Results:**

Cytotoxicity of sealers decreased gradually (*P* < 0.0001). AH26 showed the highest level of cytotoxicity (*P* < 0.001). In terms of cytotoxicity, no considerable differences were observed between the two-calcium silicate-based sealers (*P* > 0.05). AH26 showed the lowest mineralization activity (*P* < 0.0001). Among the calcium silicate-based sealers, mineralization and formation of calcium nodules were more frequently observed in the Endoseal MTA group (*P* < 0.001).

**Conclusion:**

The examined calcium silicate-based sealers had less cytotoxicity and higher mineralization activity than the resin-based sealer (AH26). There was negligible difference between the cytotoxicity of the two-calcium silicate-based, but the cell mineralization caused by Endoseal MTA was higher.

## 1. Background

Correct root canal treatment of teeth with apical periodontitis is done following the complete removal of infected pulp, while root canal preparation is followed by the establishment of apical and coronal seals [1]. The purpose of root canal obturation as the last stage of endodontic treatment is to create a gap-free environment throughout the entire root canal system to prevent recurrent infection and any communication between the internal space of the root canal and the periapical tissues [2].

Currently, in most cases, root canals are obturated by gutta-percha cones along with endodontic sealers. The main application of endodontic sealers is to fill the empty spaces between the canal walls and gutta-percha cones [3]. Sealers prevent the penetration of microorganisms and their reproduction inside the root canal by properly sealing in the entire root canal space, especially the coronal and apical areas [4]. The advantages of using sealers are not only related to filling the remaining void spaces inside the canal but also because of their antimicrobial properties and bacterial growth prevention [2].

The contact between biomaterials and tissues leads to interactions; therefore, these materials should be biocompatible and not harmful to the biological environment [3]. Sealer components may be cytotoxic to human cells, leading to inflammation and DNA damage, resulting in genome instability and increased carcinogenesis; these substances interfere with cellular elements, including lipids, proteins, and DNA which may harm the unity of the membrane according to the chemical composition of its surface [5].

Fibroblasts have many important roles, including the healing of periodontium. These cells are necessary to regenerate the firm fibrillar link between the tooth root, gingiva, and periodontal ligament [6]. Inflammatory mediators are produced by fibroblasts, immune cells found in connective tissues, in reaction to specific pathogens. By identifying pathogens, these cells recruit inflammatory cells, express proinflammatory cytokines, chemokines, and growth factors, as well as antimicrobial peptides, and have immunological qualities. To maintain pulp homeostasis and enable tissue repair and regeneration, dental pulp fibroblasts regulate their innate immune response [7].

There are various types of sealers, including eugenol-based sealers (zinc oxide eugenol), resin-based sealers (AH26 and AH plus), and calcium silicate-based sealers called “bioceramic” sealers [4]. Currently, epoxy resin-based sealers are widely used in root canal treatment. Still, several drawbacks regarding the biocompatibility of these sealers have been reported, including cytotoxic effects on fibroblasts, potential mutagenic activity, and the induction of severe inflammatory response in bone tissue. Concerns have also been raised that AH may exhibit adverse effects on the adjacent host tissues and delay the periapical healing of teeth with apical periodontitis [1].

Calcium silicate-based sealers are a new group of sealers that show a high degree of hydrophilicity and biocompatibility. Since the internal environment of the root canal is hydrophilic, water absorption and solubility of sealers are essential properties related to the stability of the sealer in the root canal. Also, due to their bioactive nature, they have a positive effect on hard tissue [8].

AH26 (Dentsplysirona, Tusla, OK, USA) is an epoxy resin-based sealer that is in powder and liquid form and was initially introduced as single obturation and was widely used due to its suitable handling characteristics. Before setting, this sealer is more toxic than when it is set, and its toxicity decreases over time.

Endoseal MTA sealer (Maruchi, Wonju, Gangwon-do, Korea) is a calcium silicate-based sealer used by injection into the root canal. It contains thickening agents, radiopacifier, calcium sulfate, calcium silicate, and calcium aluminate and has favorable characteristics such as rapid setting time, high bond strength, and bioactivity [2, 9, 10].

Ceraseal (Metabiomed, Cheongju, Chungcheongbuk-do, Korea) is a new bioceramic sealer that is used by injection, and its ingredients include calcium silicate, zirconium oxide, and thickening agents [11].

The studied properties of calcium silicate-based sealers include cell migration, which is crucial for wound healing, cell attachment, which plays an essential role in the periradicular repair process, and ion release. Additionally, research has been done on anti-inflammatory characteristics, the ability of tissue mineralization, which facilitates tissue repair, and gene expression, which promotes the repair potential. When compared to Endoseal MTA, it has been found that these properties were higher in Ceraseal sealers [12].

Considering the importance of fibroblast in the immunity system and periodontal tissue regeneration and since the application of calcium silicate sealers is increasing, the present study was conducted to examine the effect of two calcium silicate sealers compared to an epoxy resin-based sealer on the cytotoxicity and mineralization of gingival fibroblast cells.

The null hypothesis was that there would not be any significant differences in the cytotoxicity and mineralization of the sealers on gingival fibroblast cells.

## 2. Materials and Methods

### 2.1. Preparation of Cells

The human gingival fibroblast cell line was obtained from the Pasteur Institute of Iran. Human gingival fibroblast cells (type C165) were removed from the nitrogen tank and cultured in a 75 cm^2^ flask (Nunc-Denmark) containing Dulbeccos Modified Eagles Medium (DMEM) enriched with fetal bovine serum (FBS) and antibiotics. The cells were incubated in an incubator at 37°C and 5% CO_2_, and their culture medium was changed every 3 days. When the bottom of the flask was filled with cells by passage, the cells were distributed to several flasks.

### 2.2. Preparation of Sealer Extract

The sealers used in this study were AH26, Ceraseal, and Endoseal MTA. To prepare the sealer extracts, each of the sealers was prepared according to the factory instructions, and immediately before being set, they were placed in the wells of a 24-well plate (diameter 16.2 mm and height 2 mm) (1 well for each sealer). After sterilization by ultraviolet light, they were placed in an incubator for 72 hr. Then 2.5 ml of DMEM culture medium containing antibiotics without FBS was added to each well, and the plate was kept in an incubator at 37°C and 5% CO_2_ for 48 hr. After this period, the culture medium on each material was transferred to test tubes, and 10% FBS was added.

### 2.3. Inspection of Cytotoxicity

After performing several cell passages and ensuring their normal proliferation, the cells were separated from the culture flask with trypsin. The viability of the culture medium containing fibroblast was assessed with trypan blue solution and moved to a 96-well culture plate (cells/0.5 ml/well 8,000) without any toxic substances. Subsequently, the plate was placed in the incubator for 24 hr under the aforementioned standard conditions for the cells to be seeded on the plate. The cells were then inspected after 24, 48, 72, and 120 hr. By the end of the intended time interval, the cell viability percentage was evaluated by the Methyl-Thiazolyl-Tetrazolium (MTT) (3-(4,5-dimethyl-2-thiazolyl)-2,5-diphenyl-2H-tetrazolium bromide) test [13].

### 2.4. MTT Testing Method

At the end of the predetermined proximity time of cells with the sealer extract, 10 *µ*l of MTT solution was added to each of the wells of the plate and placed in the incubator for 2–4 hr. The culture medium on the cells was drained, and 50 *µ*l of dimethyl sulfoxide (DMSO) (Merk-Germany) was added to each well to dissolve the reduced formazan dye. The intensity of the resulting color was then evaluated by the light absorption of each well using the Elisa reader (Awareness Technology Inc.) at a wavelength of 545 nm (with a reference of 630 nm) [12, 13].

### 2.5. Alizarin Red Staining (ARS) Assay

The discs containing the sealer were moved to a conical tube containing 20 ml of fresh culture solution and placed in a standard atmosphere at 37°C with 5% CO_2_ for 7 days. Following these preparation steps, the solution was filtered by 0.2 *µ*m filters. The human gingival fibroblast cells containing culture medium were incubated in 24 wells with a density of 10,000 × 2 for 24 hr to form attachments. The mineralization activity was measured after 15 days, in which the sealer extract was changed every 3 days.

The cells were stained with 2% Alizarin solution for 20 min and then rinsed five times with sterile water. To evaluate the results quantitively, the stained cells were soaked with 10% cetylpyridinium chloride solution for 15 min, and measuring the absorbance at 560 nm was done utilizing an absorbance microplate reader [14].

### 2.6. Statistical Analysis

Prism, ver.3, software (GraphPad Software, La Jolla, CA) was used to perform statistical tests. One-way analysis of variance analysis, followed by Tukey's test, was used to determine group differences. *P*-values less than 0.05 were considered statistically significant.

## 3. Results

**Figure 1:**
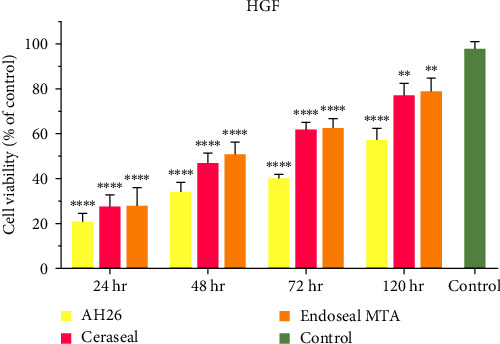
Comparison of cell viability in each sealer group with the control group over time.  ^*∗∗*^*P* < 0.01 and  ^*∗∗∗∗*^*P* < 0.0001 compared with the control group.

In due course, the cytotoxicity of sealers decreased, and the percentage of cell vitality increased. According to the experiment results, AH26 showed the highest toxicity and the lowest cell viability among the experimental groups (*P* < 0.001). There was no considerable difference between the level of cytotoxicity and cell viability between the two calcium silicate-based sealers (Ceraseal and Endoseal MTA) (*P* > 0.05) (Figure 1).

On day 0, no remarkable difference was observed in the amount of mineralization and the calcium nodule formation between the experimental and control groups. On day 15, the amount of mineralization in all sealers was significantly different in comparison with the control group and in comparison with their equivalent group on day 0 (*P* < 0.0001). On the 15th day, among the experimental groups, AH26 showed the lowest amount of mineralization compared to the Ceraseal and Endoseal MTA sealers, and this difference was significant (*P* < 0.0001).

**Figure 2:**
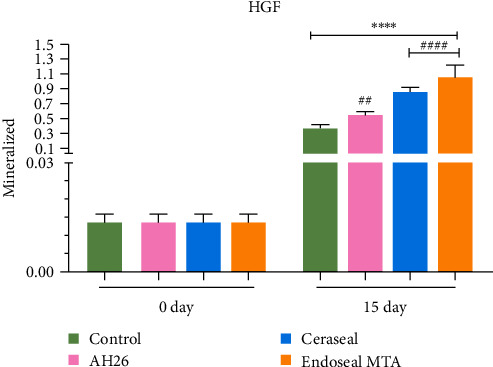
Comparison of mineralization activity in each sealer group with the control group.  ^*∗∗∗∗*^*P* < 0.0001 compared with the control group on day 0, ^##^*P* < 0.01 and ^####^*P* < 0.0001 compared with the control group on day 15.

Also, a considerable difference was detected in the amount of mineralization between Ceraseal and Endoseal MTA groups on day 15 (*P* < 0.001). The mineralization and calcium nodule formation was higher in the Endoseal MTA group (Figure 2).

## 4. Discussion

When injecting the sealer inside the canal, some reactions occur with the periapical tissue until the setting is completed [15]. Cytotoxicity emanated from sealers by releasing toxic substances to the periapical tissue, even if the sealer is not pushed out from the apex, leads to periapical destruction, bone loss, wound healing alteration, or even tooth loss [3, 16]. The biocompatibility and bioactivity of the sealer during and after the sealer's setting is related to the secretion of molecules, and the interaction of these molecules with cells as a result of using these sealers affects the cell proliferation, differentiation, and migration [17].

Resin-based sealers like AH26 have been commonly applied in endodontics. Although, formaldehyde release by these sealers leads to cytotoxicity [18]. Previous studies have shown that the maximum toxicity of this sealer is in the first 24 hr, and the toxicity of this sealer decreases as the sealer sets [14].

Zirconium oxide is a radiopacifier in this study's two calcium silicate-based sealers. According to earlier research, zirconium oxide-containing materials promote fibroblast growth while hastening the resolution of inflammatory responses. In Endoseal sealer, aluminum is one of the elements present in high concentrations. Since animal studies have shown that aluminum has genotoxicity and toxicity effects, this element can be assumed to be the root of the sealer's cell toxicity [12].

Calcium silicate-based sealers demonstrated in vitro potential for osteoblast progenitor, bone marrow, and stem cell development, as well as for osteocementogenic gene overexpression [19]. The alkalinity of calcium silicate sealers strengthens the biocompatibility and antimicrobial properties of the sealers and prevents the dissolution of minerals by neutralizing the lactic acid produced by osteoclasts [13].

Evaluation of cell viability after exposure to toxicants is determined by MTT assay, which is a water-soluble tetrazolium salt that will turn to an insoluble purple formazan after the cleavage of the tetrazolium ring by succinate dehydrogenase found in the mitochondria. The formazan cannot pass through the cell membrane and thus aggregates in normal cells [20]. ARS is a method for investigating cell mineralization activity that has been utilized to evaluate calcium-rich sediments in a growth medium [21].

Based on the results of the present study, the null hypothesis is rejected since the both bioceramic sealers (Ceraseal and Endoseal MTA) had less cytotoxicity and more mineralization than resin sealer (AH26) on gingival fibroblast cells.

Regarding cytotoxicity, there was no notable difference between Ceraseal and Endoseal MTA. In addition, the cytotoxicity of all sealers decreased over time.

Lim et al. [22] noticed considerably more viable cells in the Endoseal group on day 14 by comparing the cell viability in the Endoseal and AH plus groups, which is in line with the results of the present study. Through inspection of cell viability using the MTT assay methods conducted by Park et al. [23], the AH plus group demonstrated lower cell viability than the Ceraseal and Endoseal TCS group throughout the study, which is similar to the findings of the current study.

Among the evaluated sealers by Lee et al. [3], including Endoseal MTA and AH plus, the lowest cell viability during the study was for the resin-based sealer, which is the same as in the present investigation. Although, in contrast to the current experiment, cell viability in the AH plus and Endoseal MTA groups significantly decreased during the study; also, in a study published by Oh et al. [15], ceramic sealers had the highest cell viability compared to resin-based sealers. Similar to the previous and present studies, the findings demonstrate that calcium silicate-based sealers are more biocompatible than epoxy resin-based sealers. However, da Silva et al. [24] concluded that both Endoseal and AH plus are biocompatible.

In the study conducted by Park et al. [23], Ceraseal and Endoseal TCS showed a considerably higher absorption and cell viability than the control group on the 7th day. Cell viability increased throughout the experiment, as in the current study.

In the current study, in terms of the calcium nodules formation and cell mineralization activity, calcium silicate-based sealers performed more desirable than resin-based sealers (AH26) due to the calcium ions release. Among calcium silicate-based sealers, Endoseal MTA had higher activity than Ceraseal; in the study of Lopéz-García et al. [12], higher cell viability and mineralization capacity of Endosequence BC and Ceraseal sealers than Endoseal MTA were reported. Seo et al. [13] observed that Endoseal MTA, Endosequence BC, and BioRoot RCS formed more mineralized nodules than AH plus. Also, Oh et al. [15] found that AH plus resin-based sealer showed less staining after performing ARS assay than Ceraseal and Endoseal TCS. The findings of a study conducted by Santos et al. [19] showed more significant calcium nodule formation by calcium silicate-based sealers compared to AH plus. These findings are in line with the current study which mineralization occurred more in the calcium silicate-based sealers group than in epoxy resin-based sealers.

Clinical investigations have demonstrated that Endosequence BC sealer exposure enhances the deposition of hard tissue in human periodontal ligament stem cells [25]. The release of calcium and subsequent production of hydroxyapatite by Totalfill BC Hiflow and Totalfill BC sealers also enhance biological sealing [26].

In addition, the increase in cell proliferation and mineralization in the presence of pulp stem cells by BioRoot RCS and the induction of cell differentiation into odontoblast-like cells have been observed in iRoot sp. Also, using Endoseal MTA led to the biomineralization of dentin tubules [27, 28].

By reviewing the findings of the current study and other studies, it is evident that most studies emphasize the more desirable biocompatibility of the calcium silicate-based sealers in comparison with the epoxy resin-based sealers.

## 5. Conclusion

The findings show calcium silicate-based sealers (Endoseal MTA and Ceraseal) have less cytotoxicity and more mineralization activity than AH26. There is no considerable difference between the cytotoxicity of the two calcium silicate-based sealers, but the cell mineralization induced by Endoseal MTA was higher.

## Data Availability

The data used to support the findings of this study are available from the corresponding author upon request.
