# Correction to “Cytotoxicity and Mineralization Activity of Calcium Silicate‐Based Root Canal Sealers Compared to Conventional Resin‐Based Sealer in Human Gingival Fibroblast Cells”

**DOI:** 10.1155/ijod/9836789

**Published:** 2026-07-23

M. Shokrzadeh, F. S. Motafeghi, A. Lotfizadeh, M. Ghorbani, and A. Haddadi Kohsar, “Cytotoxicity and Mineralization Activity of Calcium Silicate‐Based Root Canal Sealers Compared to Conventional Resin‐Based Sealer in Human Gingival Fibroblast Cells,” *International Journal of Dentistry*, 2023, 4376579, https://doi.org/10.1155/2023/4376579.

In the article, clarification was missing in Sections 2.2 and 2.3 with regard to the number of biological and technical replicates. The authors have provided an updated version of these sections, which can be found below:

## 2.2. Preparation of Sealer Extract

The sealers used in this study were AH26, Ceraseal, and Endoseal MTA. To prepare the sealer extracts, each sealer was prepared according to the manufacturers’ instructions and, immediately before setting, was placed separately in designated wells of a sterile 24‐well plate (diameter 16.2 mm, height 2 mm) for initial setting and extract preparation. After ultraviolet sterilization, the materials were incubated for 72 h. Subsequently, 2.5 mL of DMEM culture medium containing antibiotics without FBS was added to each well, and the plate was maintained at 37°C in a humidified atmosphere containing 5% CO_2_ for 48 h. After this incubation period, the conditioned media from each material were collected into sterile test tubes and supplemented with 10% FBS to obtain the sealer extracts used for subsequent cellular assays.

## 2.3. Inspection of Cytotoxicity

Human gingival fibroblast (HGF) cells were cultured in Dulbecco’s Modified Eagle Medium (DMEM) supplemented with fetal bovine serum and antibiotics under standard incubation conditions (37°C, 5% CO_2_). The cells were seeded into 96‐well plates at a density of 8000 cells/0.5 mL/well. For cytotoxicity evaluation, prepared sealer extracts were applied to the cultured HGF cells. Each experimental condition was evaluated using triplicate wells (*n* = 3) at each investigated time point (24, 48, 72, and 120 h). Cell viability was assessed using the MTT assay according to the manufacturer’s protocol. Absorbance values were measured using a microplate reader at the designated wavelength.

Data were expressed as mean ± standard deviation (SD). Statistical analysis was performed using one‐way ANOVA followed by Tukey’s post hoc test, and the significance level was set at *p*  < 0.05.

We apologize for these errors.

